# Advancing ‘real-world’ trials that take account of social context and human volition

**DOI:** 10.1186/s13063-017-2286-8

**Published:** 2017-11-10

**Authors:** Anders Blædel Gottlieb Hansen, Allan Jones

**Affiliations:** 1 Strategic Research and Development Support, Metropolitan University College, Tagensvej 18, Copenhagen N, 2200 Denmark; 2 Bachelor’s Degree in Global Nutrition and Health, Faculty of Health and Technology, Metropolitan University College, Sigurdsgade 26, Copenhagen N, 2200 Denmark

**Keywords:** Complex interventions, External validity, Research funding

## Abstract

**Background:**

The recent paper in *Trials* by Porter and colleagues highlights the utility of applying a *critical realism* approach in randomised trials, an approach central to the Medical Research Council’s (MRC) Framework for the Development and Evaluation of Complex Healthcare Interventions. The MRC framework offers a pragmatic step towards a more open systems approach that bridges randomised evaluation with social context and human agency in an effort to improve the generalisability of trial outcomes.

**Main body:**

The MRC framework has contributed to the proliferation of a more open systems approach in health research; however, the broader acceptance of the realist approach to health research does not seem to be emulated by norms in research fund allocation, which largely prioritises laboratory-based research.

**Conclusion:**

This commentary is simply a plea, to those who make the strategic decisions regarding allocation of research funding, to support all phases of health intervention research in complex systems that contribute to the development of effective, translational and sustainable interventions in the promotion of health.

## Background

The dissemination over a decade ago of the Medical Research Council’s (MRC) Framework for the Development and Evaluation of Complex Healthcare Interventions [1], and appeals by researchers for more research to be conducted on interventions in complex systems [2] has contributed to the proliferation of research in more open systems, i.e. in ‘real-world’ settings (for an example, see the INCLUSIVE study – [3]). The MRC framework advocates a mixed-method approach, including: qualitative research – such as process evaluation of implementation of an intervention and of contextual factors that could lead to variability in outcomes [4]; causal modelling approaches, that can provide information on the process and outcome of an intervention and that may lead to design changes prior to implementation, e.g. [5]; and a range of experimental methods for evaluating effectiveness, with preference given to randomised designs (randomised controlled trials – RCTs), wherever possible or appropriate, for optimisation of internal validity [6]. The mixed-method approach is an attempt to supplement the randomised trial with procedures embedded in *realism* that take the complexity of social context and human volition into account, and to shift the focus from identifying causality to explaining causality, or in other words, to explaining how intervention components interact with different populations and in different contexts [7]. Moore and Evans [8] demonstrate the importance of context by describing how an intervention nearly two decades ago, that effectively reduced smoking in young people by prohibiting smoking on school premises and thereby challenging norms surrounding smoking behaviour, may not prove as effective today. Norms surrounding smoking that existed 15–20 years ago (e.g. high prevalence rates and social acceptance) have changed, and consequently, so have the mechanisms that predict smoking behaviour in young people [8].

The combination of positivist and realist approaches found in the MRC framework, which Porter et al. [7] and others [9] refer to as *realist RCTs*, has given rise to debate [7, 9–13]. Central to the debate is whether randomised and realist evaluation can be consolidated. While critics argue that the realist RCT design found in the MRC framework does not fully take complexity into account [12], the MRC framework does offer a pragmatic step towards a more open systems (real-world) approach that bridges, to a degree, randomised evaluation with social context and human agency [14]. The recent paper in *Trials* by Porter and colleagues highlighting the utility of applying a *critical realism* approach [7] and the ongoing debate exploring the utility between realist and randomised evaluations [9, 12] are welcome and important steps towards the development and refinement of a usable open systems approach.

### A plea for more research funding aimed at supporting health interventions in complex systems

While incremental steps are being made as evidenced by the MRC complex framework, the broader adoption of a more open systems approach in health research does not seem to have penetrated the orthodoxy of applying a traditional positivist epistemological and ontological (bio-medical) approach to evaluating research and, by extension, to deciding how research funds are allocated [15, 16]. For example, an overview of research-fund allocation from the Medical Research Council, The Wellcome Trust, British Heart Foundation, and Cancer Research UK, showed that across the four research-funding agencies, 85% of funds on average were allocated to laboratory-based research [17]. A recent review of funding trends within diabetes research illustrated that the proportion of funded studies that included social context and human volition was small compared to the proportion of funded bio-medical studies, with an estimated mean ratio of 17:1 [16]. The uptake of a non-linear, or real-world conception of causality by funding agencies seems, therefore, to be a slow burn. There is no doubt that the closed linear system approach, so pervasive in bio-medical and health research, has been effective in solving health-related and other issues. However, researchers, including those within bio-medicine, are increasingly asking whether the documented efficacy of an intervention, gained from identifying causality in a closed part of a larger or open system (traditional reductionist approach), is robust enough to reproduce the recorded effects reliably in real-world settings [18, 19]; or if successionist models of causality alone are adequate in solving the complex health issues of our time [20–23].

It is estimated that as much as 85% of research investment can be categorised as waste (i.e. not benefitting society/patients) due largely to avoidable weaknesses in research design and production [24–27]. Lack of external validity in the experimental research designs employed is one of the main reasons why laboratory-based and randomised clinical trial outcomes often fail to translate into benefits for the patient, as the robustness of probabilities in different contexts (what works best for whom and in which setting) is uncertain [12, 18, 19, 21, 28]. Laboratory and clinical trials that do not take higher-level concepts, such as social context and human volition, into account are, therefore, vulnerable when attempting to replicate intervention effects in real-world settings. As stated by Rothwell: *“Government expenditure should provide value for money, and medical research is no exception”* [17].

The MRC framework is an attempt to increase the external validity of evaluations while still preserving internal validity. The availability of more research funds that support health interventions and practice-orientated research in complex systems may result in more robust and sustainable interventions and lead to reduced waste in research investment.

## Conclusion

Since the introduction of the MRC framework, complex interventions are increasingly being developed in an attempt to solve the continually expanding burden of health-related issues. This commentary is, therefore, a plea to policy-makers, and those who make the strategic decisions regarding allocation of research funding, to support to a greater degree all phases of real-world trials that take into account social context and human volition as advocated by the MRC framework, rather than to automatically allocate research funds on the basis of received wisdom.

## Abbreviations

MRC: Medical Research Council
RCTs: Randomised controlled trials
